# A Possible Explanation for the Low Penetrance of Pathogenic KCNE1 Variants in Long QT Syndrome Type 5

**DOI:** 10.3390/ph15121550

**Published:** 2022-12-13

**Authors:** Szilvia Déri, Teodóra Hartai, László Virág, Norbert Jost, Alain J. Labro, András Varró, István Baczkó, Stanley Nattel, Balázs Ördög

**Affiliations:** 1 Department of Pharmacology and Pharmacotherapy, University of Szeged, 6720 Szeged, Hungary; 2 ELKH-SZTE Research Group for Cardiovascular Pharmacology, 6720 Szeged, Hungary; 3 Department of Basic and Applied Medical Sciences, University of Ghent, 9000 Ghent, Belgium; 4 Department of Medicine, Montreal Heart Institute, Université de Montréal, Montréal, QC H3T 1J4, Canada

**Keywords:** LQT5, KCNE, KCNQ1, potassium channel, cardiac arrhythmia

## Abstract

Long QT syndrome (LQTS) is an inherited cardiac rhythm disorder associated with increased incidence of cardiac arrhythmias and sudden death. LQTS type 5 (LQT5) is caused by dominant mutant variants of KCNE1, a regulatory subunit of the voltage-gated ion channels generating the cardiac potassium current I_Ks_. While mutant LQT5 KCNE1 variants are known to inhibit I_Ks_ amplitudes in heterologous expression systems, cardiomyocytes from a transgenic rabbit LQT5 model displayed unchanged I_Ks_ amplitudes, pointing towards the critical role of additional factors in the development of the LQT5 phenotype in vivo. In this study, we demonstrate that KCNE3, a candidate regulatory subunit of I_Ks_ channels minimizes the inhibitory effects of LQT5 KCNE1 variants on I_Ks_ amplitudes, while current deactivation is accelerated. Such changes recapitulate I_Ks_ properties observed in LQT5 transgenic rabbits. We show that KCNE3 accomplishes this by displacing the KCNE1 subunit within the I_Ks_ ion channel complex, as evidenced by a dedicated biophysical assay. These findings depict KCNE3 as an integral part of the I_Ks_ channel complex that regulates I_Ks_ function in cardiomyocytes and modifies the development of the LQT5 phenotype.

## 1. Introduction

Long QT syndrome (LQTS) is a primary cardiac rhythm disorder characterized by the prolongation of the QT interval on the electrocardiogram and by the increased incidence of ventricular arrhythmias and sudden cardiac death (SCD) [1]. To date, 17 LQTS subtypes have been identified and classified by the underlying genotype. The three major subtypes including LQT1, LQT2 and LQT3 are accountable for 70% of the cases [2]. LQT1 is caused by loss of function variants of the KCNQ1 gene, encoding the pore-forming subunit of ion channels mediating the cardiac slow-delayed rectifier current I_Ks_. I_Ks_ contributes to phase 3 of the cardiac action potential (AP) and is an important component of the repolarization reserve [3,4]. Among the much less prevalent subtypes, LQTS type 5 (LQT5) is caused by dominant negative, loss of function mutations in the KCNE1 gene encoding an important regulatory subunit of I_Ks_ channels [5,6].

Penetrance of LQTS mutations is incomplete in general and varies with LQTS type [7,8]. Approximately 75% of patients with LQT1–3 genotype are symptomatic [9]. In sharp contrast, the overall penetrance of LQT5 mutations was as low as 20% in a recent international multicenter study involving 89 probands and their 140 genotype positive family members [10]. It appears, therefore, that whilst both LQT1 and LQT5 are associated with loss-of-function variants of the genes needed for I_Ks_ function, penetrance of LQT5 variants is markedly lower compared to LQT1. Importantly, even though clinical risk stratification in LQTS is primarily based on manifested symptoms, concealed subclinical mutation carriers are at substantial risk of arrhythmic events, particularly when exposed to QT-prolonging drugs [11]. The mechanisms underlying the relatively low penetrance of LQT5 mutations have not been explored.

A transgenic rabbit model of LQT5 has been developed recently and provided vital insights into the LQT5 phenotype [12,13]. In line with the clinical manifestations of the disease, LQT5 rabbits displayed increased arrhythmia propensity in response to QT-prolonging drugs compared to wild type animals, but showed no manifested QT-prolongation under baseline conditions. The LQT5 rabbit model is based on the cardiac-specific over-expression of the pathogenic LQT5 KCNE1 variant G52R-KCNE1 [14]. G52R-KCNE1 has a strong negative effect on current amplitudes when co-expressed heterologously with KCNQ1 in the presence of wild type KCNE1 (WT-KCNE1) [14]. Quite surprisingly, I_Ks_ amplitudes were not different in native ventricular cardiomyocytes isolated from LQT5 compared to WT rabbits. In fact, only the time course of I_Ks_ deactivation was accelerated in cells from LQT5 animals [12]. These apparently contradictory findings suggest that KCNE1 may not be the sole regulatory subunit governing I_Ks_ function.

In particular, members of the KCNE gene family (KCNE2–KCNE5) are also expressed in the heart [15,16,17] and have been shown to interact with KCNQ1 channels in vitro [16,18]. KCNE3 has been shown to modulate KCNQ1 function and render KCNQ1-based channel constitutively open [18,19]. Furthermore, ectopic over-expression of KCNE3 abbreviated the action potential durations (APD) both in isolated rabbit cardiomyocytes ex vivo and in guinea pig hearts in vivo, possibly due to an interaction with the delayed rectifier currents [20]. These findings make KCNE3 a possible candidate I_Ks_ regulatory subunit to account for the LQT5 phenotype.

In this study, therefore, we studied the interactions of KCNE3 with channels based on KCNQ1 and different combinations of WT and LQT5 mutant variants of KCNE1 by the heterologous co-expression of these ion channel subunits and by the subsequent characterization of whole cell transmembrane currents in patch clamp experiments. The LQT5 KCNE1 variants G52R-KCNE1 and D76N-KCNE1 with well-documented dominant negative effects were included [14,21]. Physical interactions between putative I_Ks_ subunits were assessed by the NanoBiT split reporter assay for protein–protein interactions.

## 2. Results

### 2.1. General Current Phenotype

**Figure 1:**
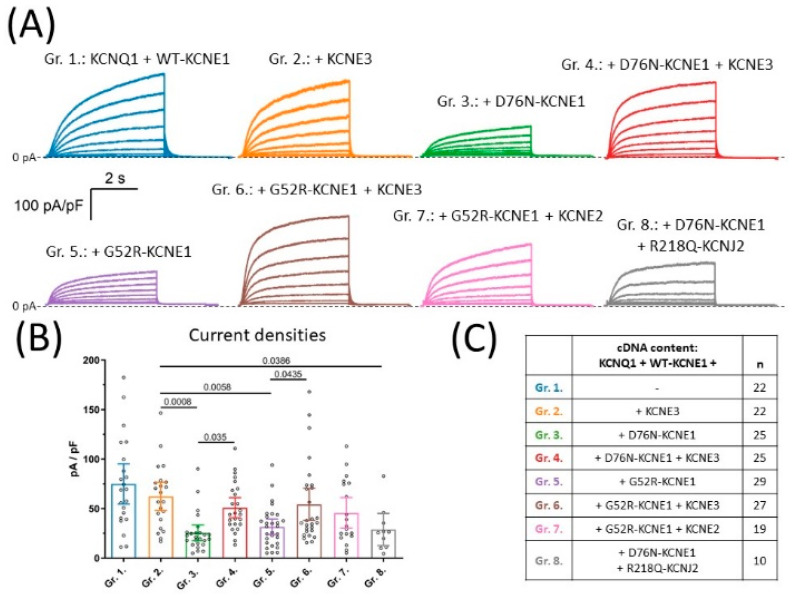
Representative current recordings (**A**) and mean ±95% CI current densities (**B**) observed in cells co-expressing KCNQ1 and WT-KCNE1 together with different combinations of LQT5 mutant KCNE1, KCNE2 or KCNE3, as indicated in (**C**), where *‘n’* means number of experiments. Average current densities were statistically compared by one-way ANOVA followed by Holm–Sidak’s post-hoc tests. Multiplicity-adjusted *p* values are shown where difference between group means was considered significant (*p* < 0.05).

All products of the members of the KCNE gene family (KCNE1 to KCNE5) modulate the biophysical properties of the KCNQ1-based current in vitro. For example, KCNQ1 alone conducts a current with rapid activation and low conductance, a slowly activating current in the presence of KCNE1 [6], while KCNE3 renders KCNQ1 constitutively active [19]. In this study, KCNQ1 and KCNE1 were co-expressed with different combinations of LQT5 KCNE1 variants, KCNE2 and KCNE3 (Figure 1C). All whole-cell current recordings obtained in this study (*n* = 179) replicated the general phenotype of the current produced by KCNQ1/KCNE1 complexes (i.e., slow time-dependent activation, Figure 1A). To characterize this general current phenotype, current traces elicited by a 50-mV test pulse were fitted by a bi-exponential function and time constants of the fast and slow exponential components were statistically compared in each group. Group means of time constants were in the range of 270–370 ms and 2800–4800 ms for the fast and slow exponential component, respectively, and were not significantly different between experimental groups (*p* > 0.05, Figure S1 (Supplementary Materials)).

### 2.2. KCNE3 Rescues Current Densities in the LQT5 Context

LQT5 KCNE1 variants, such as D76N- and G52R-KCNE1, suppress current amplitudes in a dominant negative way when expressed together with KCNQ1 and WT-KCNE1. In this study, this situation is represented by experimental groups 3 and 5, respectively (Figure 1C). Average current density was 25.5 pA/pF (95% CI [17.6–33.4], *n =* 25) in group 3 and 31.7 pA/pF, 95% CI [23.8–39.5], *n =* 29) in group 5, which both were significantly lower (*p* < 0.0001 for both comparisons) compared to group 1 (74.9 pA/pF (95% CI [54.7–95.2], *n =* 22, Figure 1B). These data confirm the dominant negative effect of the D76N- and G52R-KCNE1 variants over WT-KCNE1 on I_Ks_ current amplitudes, when I_Ks_ channels are composed of KCNQ1 and KCNE1 only.

Clinical observations about the relatively low penetrance of LQT5 KCNE1 variants suggest, however, that their inhibitory effect on I_Ks_ function is not well-represented by the results of isolated co-expression of KCNQ1/KCNE1-WT and KCNE1-mutant; one explanation could be an effect of regulatory subunits other than KCNE1 that are also present in vivo. To explore this possibility, KCNE3 was co-expressed in combinations with KCNQ1 and various KCNE1 variants in groups 2, 4 and 6, representing the normal and the heterozygous D76N- and G52R-KCNE1 LQT5 genotypes, respectively (Figure 1C). On the normal genetic background, KCNE3 had no effect on current density, as shown by average current densities in group 2 (62.33 pA/pF, 95% CI [48.2–76.5], *n =* 22) compared to group 1 (*p* = 0.455, Figure 1B). The inhibitory effects of D76N- and G52R-KCNE1 were still obvious when comparing the mean current densities of group 3 and group 5 to that of the KCNE3-containing normal genotype represented by group 2 (*p* = 0.0008 and *p* = 0.0058, respectively, Figure 1B). KCNE3, on the other hand, activated KCNQ1-based currents in the LQT5 context. Current densities were significantly increased when KCNE3 was added to the D76N-KCNE1 and G52R-KCNE1-based genetic background in group 4 (51 pA/pF, 95% CI [40.9–61.1], *n =* 25) and in group 6 (54.3 pA/pF, 95% CI [38.2–70.5], *n =* 27), in comparison to group 3 (*p* = 0.0035) and to group 5 (*p* = 0.0435), representing the same LQT5 genotypes without KCNE3, respectively (Figure 1B). Furthermore, no statistically significant difference was found when comparing average current densities in groups with subunit compositions that included KCNE3 in the presence (group 4 and group 6) or absence (group 2) of LQT5 KCNE1 variants (*p* = 0.455 for both comparisons, Figure 1B). These findings indicate that KCNE3 rescues I_Ks_ from the inhibitory effect of the LQT5 KCNE1 variants from two distinct LQT5 genetic backgrounds.

Since close relatives of KCNE3 are also expressed in the heart, we thought about exploring whether the rescue effect is unique to KCNE3 or can also occur in the presence of other KCNE subunits. To this end, KCNE2 was co-expressed in the presence of G52R-KCNE1 in group 7 (Figure 1C). Average current density in group 7 was 45.7 pA/pF (95% CI [30.4–61.2], *n =* 19), which was statistically indistinguishable from group 5 (*p* = 0.4207, Figure 1B). Our experiments, therefore, did not provide evidence for a rescue effect of KCNE2 on the G52R-KCNE1 LQT5 genetic background, which possibly should be investigated in dedicated studies.

Finally, since transfection mixtures for groups 4, 6 and 7 contained the largest number of different plasmid DNA constructs, in order to rule out any possible artefacts originating from this experimental condition, the rescue of I_Ks_ amplitudes was attempted by including the non-conductive KCNJ2 variant R218Q-KCNJ2 in group 8 (Figure 1C). KCNJ2, an inward rectifier potassium channel subunit, belongs to a different structural class of potassium channels and does not interact with KCNQ1 directly. In addition, the R218Q-KCNJ2 variant has zero conductance [22], therefore its presence does not interfere with the patch clamp recording of KCNQ1-based currents. Mean current density was 28.2 pA/pF (95% CI [12.7–45.1], *n =* 10) in this group, significantly lower than in group 2 (*p* = 0.0386, Figure 1B). The rescue effect of KCNE3 on I_Ks_ amplitudes is therefore not due to an artefact caused by the varying number of plasmid DNA constructs in transfections.

### 2.3. Deactivation Kinetics Are Accelerated in the Presence of LQT5 KCNE1 Variants

**Figure 2:**
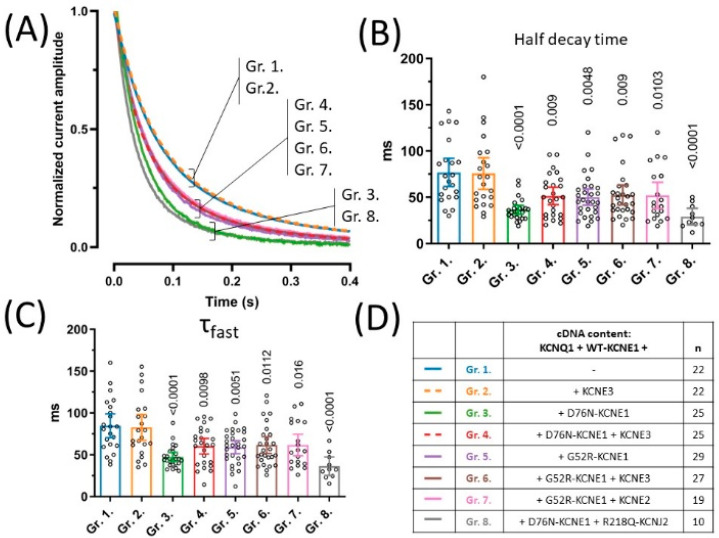
Deactivation kinetics were assessed by characterizing the kinetics of current decay of tail currents observed at −40 mV following a 5 s long activating pulse from −80 mV to 50 mV (**A**). Half decay times of tail currents were defined as time from tail current peak to 50% decay (**B**), whereas time constant of the fast exponential component were extracted from non-linear curve fits of tail current traces (**C**). Symbols represent individual data points; bar graphs represent mean ±95% CI. Group means were statistically compared to that of Group 2. by one-way ANOVA followed by Holm–Sidak’s post-hoc tests. Multiplicity-adjusted *p* values are shown where difference between group means was considered significant (*p* < 0.05). cDNA content of transfections are indicated in (**D**), where *‘n’* means number of experiments.

The rate of current deactivation, expressed by the deactivation time-constant, is an important determinant of I_Ks_ function. We studied this current-property on the same recordings that were used for the assessment of current densities (Figure 2D), by analyzing the time course of decay of tail currents recorded at −40 mV following a 50 mV activating pulse (Figure 2A). Time constants of tail current decay were extracted in two independent ways. First, half-decay times of deactivation, corresponding to the time of 50% decay of tail current amplitude were determined (Figure 2B). KCNE3 had no effect on half decay times of channels based on KCNQ1 and WT-KCNE1, as indicated by 77 ms (95% CI [61.8–92.1], *n =* 22) and 75.7 ms (95% CI [58.6–92.8], *n =* 22) average half decay times in groups 1 and 2, respectively (*p* = 0.8746, Figure 2B). Half decay times in each other experimental group, however, were significantly decreased when compared to group 2 (Figure 2B). This indicates an accelerated deactivation kinetics in the presence of both D76N- and G52R-KCNE1 LQT5 KCNE1 alleles, irrespective of the presence of KCNE3. The latter finding was reproduced by the second method to assess deactivation kinetics, where time constants of current decay were extracted from the non-linear curve fitting of tail currents. The time constants of the fast exponential component (*τ*fast, Figure 2C) were significantly decreased in groups 3–8, compared to group 2. (Figure 2C), while the time constants of the slow exponential component were not different (*p* = 0.1548, Figure S2).

### 2.4. Activation Kinetics Are Not Affected by LQT5 KCNE1 Variants or KCNE3

**Figure 3:**
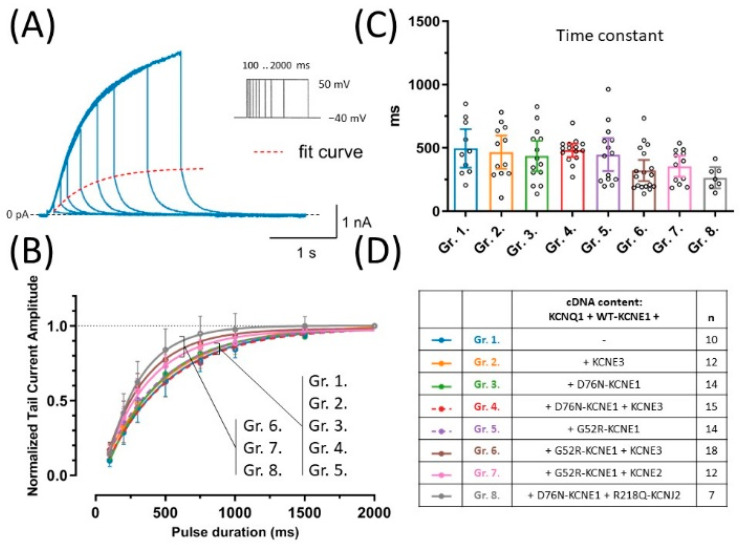
Activation kinetics were assessed by the envelope of tail currents protocol, which consisted of a series of 50 mV test pulses of increasing duration. A representative recording of an experiment in group 1 (blue), the fitted curve (dashed red) and the voltage clamp protocol are shown (**A**). Normalized peak tail currents were plotted against test pulse duration (**B**), time constants were extracted from non-linear curve fits (**C**). Symbols and error bars represent mean values ±95% CI, continuous lines represent curve fits to average values. One-way ANOVA showed no statistically significant difference between group means. cDNA content of transfection mixtures are indicated in (**D**), where *‘n’* means number of experiments.

To assess whether any of the LQT5 KCNE1 variants or KCNE3 has an effect on the time course of activation of KCNQ1 and WT-KCNE1-based currents, the envelope of tail currents protocol was applied, in which tail currents are recorded after activating pulses of variable durations (Figure 3A). Time constants were extracted from the exponential fit of peak tail currents plotted against test pulse duration (Figure 3B). Average time constants of activation were not different (*p* = 0.0884) and hence neither the LQT5 KCNE1 variants, nor KCNE3 had any detectable effect on activation kinetics (Figure 3C).

### 2.5. D76N-KCNE1 Shifts Voltage Dependence of Activation Irrespectively of the Presence of KCNE3

**Figure 4:**
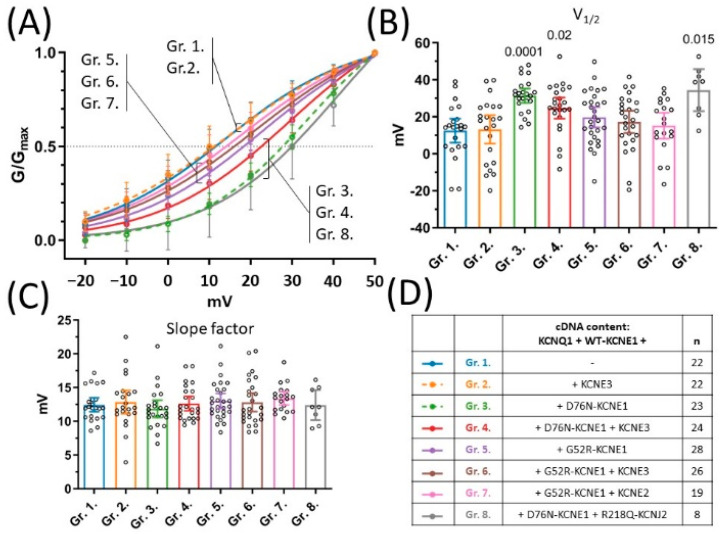
Voltage-dependence of steady-state activation. Steady-state activation curves as represented by mean normalized conductance ±95% CI values and non-linear curve fits (**A**). Mean ±95% CI half-maximal activation voltages (**B**) and mean ±95% CI slope factors (**C**) were statistically compared by one-way ANOVA followed by Holm–Sidak’s post-hoc tests. Multiplicity-adjusted *p* values are shown where difference between group means was considered significant (*p* < 0.05). cDNA content of transfection mixtures are indicated in (**D**), where ‘*n*’ means number of experiments.

Voltage dependence of activation was characterized by extracting voltage of half maximal activation (V1/2) and the slope factor from non-linear curve fit of steady-state activation curves (Figure 4A). KCNE3 had no effect on V1/2 of the current produced by KCNQ1 and WT-KCNE1, as indicated by the average V1/2 values in group 2 (13.2 mV 95% CI [5.5–20.9], *n =* 22) compared to group 1 (12.6 mV 95% CI [6.0–19.1], *n =* 22, *p* = 0.88) (Figure 4B). Similarly, there was no difference in average V1/2 values observed in the presence of the G52R-KCNE1 allele, expressed in experimental groups 5, 6 and 7 (Figure 4D), compared to the KCNQ1/WT-KCNE1 subunit composition represented by group 1 (*p* = 0.2658, 0.5763 and 0.8013, respectively, Figure 4B). The D76N-KCNE1 variant, on the other hand, shifted V1/2 values markedly to the positive direction, as indicated by the significantly increased average V1/2 values in groups 3, 4, and 8 (Figure 4D), compared to group 1 (*p* = 0.0001, 0.02 and 0.015, respectively, Figure 4B). There was no statistical difference between the slope factors among all experimental groups (*p* = 0.8, Figure 4C). D76N-KCNE1, but not G52R-KCNE1, therefore, shifts the voltage dependence of current activation in the positive direction, contributing to the loss-of-function nature of the D76N-KCNE1 allele, which phenomenon is similarly detected in the presence and in the absence of KCNE3.

In summary, patch clamp experiments showed that KCNE3 has no effect on currents driven by ion channels composed of KCNQ1 and WT-KCNE1. KCNE3 nevertheless prevents the inhibitory effect of both D76N-KCNE1 and G52R-KCNE1 variants with respect to the densities of KCNQ1/WT-KCNE1-based currents. However, the rescue effect of KCNE3 is incomplete. The accelerated deactivation kinetics caused by both LQT5 KCNE1 variants and the right shift of steady-state activation in the presence of the D76N-KCNE1 variant remained unaffected by the co-expression of KCNE3. The patch clamp data reported in this study show that in the presence of KCNE3 and LQT5 KCNE1 variants, the KCNQ1 current displays a unique blend of the properties of ion channels with different subunit combinations, including features of the KCNQ1 current on the LQT5 genetic background (accelerated deactivation) and properties of KCNQ1/WT-KCNE1 channels (amplitude and activation kinetics) as well.

### 2.6. KCNE3 Replaces KCNE1 in the I_Ks_ Channel Complex

**Figure 5:**
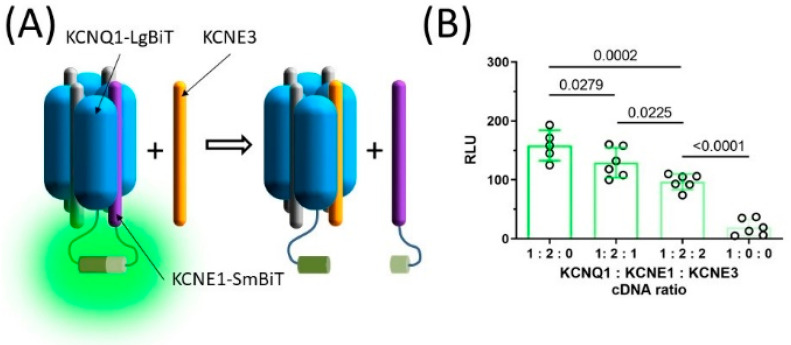
KCNE3 shifts subunit stoichiometry of the I_Ks_ channel complex. The large (LgBiT) and the small (SmBiT) fragment of the split NanoLuc luciferase were fused the C-termini of KCNQ1 and KCNE1, resulting in KCNQ1-LgBiT and KCNE1-SmBiT. The LgBiT and SmBiT fragments complement each other and produce bioluminescence when in the proximity to each other (**A**). Average ±95% CI relative bioluminescence values (RLU) observed in the presence of varying KCNQ1-LgBiT, KCNE1-SmBiT and KCNE3 ratio (**B**). Group means were statistically compared by one-way ANOVA followed by Holm–Sidak’s post-hoc tests.

Since the macroscopic whole cell currents studied here represent the activity of the entire population of ion channels that are present in the cell membrane, whether KCNE3 and KCNE1 co-assemble in the same ion channel complex or distribute in different ion channel populations remains elusive. To gain insights into this question, an experiment based on the NanoBiT protein: protein interaction assay was conducted. The NanoBiT assay relies on large and small fragments of the Nanoluc luciferase (LgBiT and SmBiT, respectively), which complement each other and form the bioluminescent enzyme when in the vicinity of each other [23]. This capacity of the NanoBiT assay was used to test whether KCNE3 has any effect on the interaction between KCNQ1 and KCNE1 (Figure 5A). LgBiT and SmBiT were fused to the C-termini of KCNQ1 and WT-KCNE1, respectively. The KCNQ1-LgBiT and KCNE1-SmBiT reporter constructs were co-expressed with varying amounts of KCNE3 to yield 1:2:0, 1:2:1 and 1:2:2 KCNQ1: KCNE1: KCNE3 cDNA ratio. Average relative luminescence (RLU) was 158.4 (95% CI [126.4–190.4], *n =* 5) in the absence of KCNE3 (KCNQ1: KCNE1: KCNE3 cDNA ratio 1:2:0), identified by Grubbs test (Alpha = 0.05, Figure 5B). In the presence of KCNE3 (cDNA ratio 1:2:1), average RLU was significantly smaller (129.3, 95% CI [102.7–155.9], *n =* 6, *p* = 0.0279, Figure 5B), while including equal amounts of KCNE1 and KCNE3 cDNA in the transfection mixture (cDNA ratio 1:2:2) reduced average RLU even more (96.7, 95% CI [82.7–110.6], *n =* 6, *p* = 0.0225, Figure 5B). These data indicate that in the presence of KCNE3, I_Ks_ channels contain a lower number of KCNE1 subunits on average. KCNE3 therefore lowers the distribution of KCNE1 in I_Ks_ channels, likely by replacing KCNE1 in the ion channel complex.

In summary, we found that KCNE3 prevents the inhibitory effect of LQT5 KCNE1 variants, when co-expressed heterologously. In addition, subunit compositions representing the heterogeneous LQT5 genetic background completed with KCNE3 produce currents with accelerated deactivation kinetics, thereby recapitulating I_Ks_ properties observed in the transgenic LQT5 rabbit model. KCNE3 accomplishes these effects by replacing KCNE1 within the macromolecular complex of I_Ks_ channels.

## 3. Discussion

Our study was conceived in the light of the findings on the phenotype of the transgenic rabbit model of LQT5 [12]. These rabbits showed a relatively weak phenotype at baseline, however, they were markedly more prone to have the Torsade de Pointes-type of ventricular arrhythmia (TdP) upon I_Kr_ blockade by dofetilide. This phenotype is closely in line with the clinical observations on the low penetrance of causative mutations in LQT5, producing no obvious symptoms in many cases, but increasing arrhythmia susceptibility and risk of SCD [7,8,10]. Therefore, the LQT5 transgenic rabbit model is of excellent use for the reliable, preclinical cardiac-electrophysiological safety testing of drugs [24].

Nevertheless, the characterization of the cellular phenotype in these rabbits highlighted an important area of cardiac cellular electrophysiology, where our knowledge is limited. Cardiac ion channels can be regarded as macromolecular complexes of often multiple subunits, in which a core, containing the pore that serves as passage for ions through the cell membrane, built up by the so-called pore-forming or α-subunits, is surrounded by strongly associated β-subunits, essentially shaping the ion channel function [25]. The exact subunit composition of the ion channel macromolecular complex and the stoichiometric balance of different subunits, likely to be under the dynamic influence of as yet unknown factors, is difficult to determine and is poorly understood [26]. It has recently been demonstrated that KCNE1 can possibly occupy up to four sites within the KCNQ1 complex and that single-channel conductance and voltage-dependence of activation rely on the stoichiometric ratio between KCNQ1 and KCNE1 [27]. Furthermore, the dynamic partnership between the KCNQ1-KCNE1 complex and β-subunits different from KCNE1, such as KCNE4 and KCNE2, have also been evidenced [28,29]. These results strongly support the paradigm in which I_Ks_ channels exist as dynamically regulated, structurally distinct complexes of KCNQ1 and a number of distinct regulatory subunits.

Murray et al. (2016) published voltage of half maximal activation (V1/2) values for KCNQ1-based channels containing KCNE1 in different stoichiometric ratios [27]. Notably, in our experiments, V1/2 was ~12 mV, markedly lower than that of an I_Ks_ complex saturated by 4 KCNE1 subunits (~30 mV) and well within the range of V1/2 values published for channels containing only two KCNE1 subunits [27]. This suggests that transient transfections resulted in KCNQ1 complexes that were not saturated with KCNE1, when no other β-subunit was present. The additionally co-expressed β-subunits, including the LQT5 KCNE1 variants or KCNE3 could therefore either occupy the remaining empty binding sites or could even replace KCNE1 when present in sufficient amount. While subunit stoichiometry has not been directly addressed in this study, the experiment carried out by using the NanoBiT split reporter assay provides important insight into the mechanistic bases of the effects of KCNE3. The NanoBiT assay showed that KCNE3 shifts the subunit stoichiometry of KCNQ1-based channels by lowering the average amount of KCNE1 co-assembled in the ion channel complex. Whether KCNE3 interacts with WT- and mutant KCNE1 variants similarly and the mechanism by which KCNE3 interferes with KCNE1 binding remains to be determined in further studies.

Co-transfection of plasmid DNA results in highly correlated transgene expression in the target cells, however, expression levels vary greatly from cell to cell and is under the influence of several factors including resource competition [30,31]. Furthermore, ion channel subunits may co-assemble in functional ion channel complexes in different stoichiometric ratios within the same cell. This heterogeneous ion channel population is sampled by performing patch clamp experiments on individual cells. In this experimental setting, whole-cell currents do not provide sufficient information on the functional properties of a particular subunit configuration. Due to this limitation, the molecular mechanism of the rescue effects by KCNE3 as they have been shown here, remain elusive. Such insights could be gained by studies using assays that provide information on subunit composition or engineered channels with predefined subunit stoichiometry, such as in Murray et al. (2016).

It is important to note, however, that each current recorded under these conditions, including the ones from cells that were transfected with four different ion channel subunit cDNAs, showed the general phenotype of a KCNQ1-WT-KCNE1 complex and none of them showed the phenotype of KCNQ1 alone or the constitutively active phenotype of channels composed of KCNQ1 and KCNE3. Furthermore, D76N-KCNE1, but not G52R-KCNE1, is known to right-shift steady-state activation of KCNQ1 by ~15 mV [14,21]. We found a positive shift of similar magnitude in V1/2 values in each experimental group containing D76N-KCNE1, but not with G52R-KCNE1 or KCNE3. These data provide evidence that the heterogeneous cell populations generated by transient transfections are adequately represented by the samples produced by patch clamping.

In our experiments, KCNE3 had no effect on the time course of activation of the KCNQ1 + WT-KCNE1 channels, apparently contradicting the previously published results [17]. In the study by Lundquist et al. (2006), a transgenic CHO cell line stably expressing KCNQ1 and KCNE1 was used and the KCNE3-encoding cDNA was delivered by liposome-mediated transient transfections. This approach is distinctly different from the one employed in our study where all cDNA plasmid mixtures were delivered exclusively by transfection. Different methodologies of gene delivery may result in a different copy number of the expression vector in the nuclei of the target cells, resulting in different gene expression levels, subunit composition of the expressed ion channels and ultimately altered characteristics of the macroscopic currents. 

I_Ks_ is thought to have only a limited influence on cardiac repolarization under baseline conditions but to activate at elevated heart rates, upon sympathetic stimuli or when other repolarizing currents are downregulated or blocked [32,33,34]. The unique activation and deactivation kinetics of I_Ks_ underlie its rate-dependent accumulation, whereas current activation by adrenergic signaling pathways is mediated by direct phosphorylation of the channel by PKA [35,36].

Our study provides important insights into interactions between I_Ks_ β-subunits in the context of an inherited channelopathy, LQT5. We demonstrate that the in vivo effects of the G52R LQT5 KCNE1 variant on I_Ks_ can be reproduced in vitro by co-expressing KCNE3 with the WT- and LQT5 KCNE1 variants. We show that KCNE3 modifies the effects of two different LQT5 KCNE1 mutations profoundly by preventing knock-down of the amplitude of macroscopic currents. KCNE3 is able to achieve this by distributing in the same ion channel complexes with KCNE1, as indicated by the experimental results from our dedicated biophysical assay. These findings strengthen the notion of the heteromeric nature of I_Ks_ channels consisting of not only KCNQ1 and KCNE1, but other structurally related auxiliary subunits as well. Subunit composition and stoichiometry is an important determinant of not only the functional properties, but also the pharmacological profile of I_Ks_ channels [37,38]. Therefore, this potential diversity of possible combinations of protein subunits that co-assemble in I_Ks_ channels must be taken into account during the design of studies that employ the heterologous expression of I_Ks_ channels and the interpretation of experimental results produced by them.

Importantly, the I_Ks_ channel subunit configuration that contains KCNE3 together with LQT5 KCNE1 variants corresponds to that of asymptomatic or ‘silent’ LQT5 patients represented by LQT5 rabbits. Why and how symptoms manifest in some, but not in all LQT5 mutation carriers remains elusive. Nevertheless, the dynamic subunit stoichiometry of I_Ks_ can be considered as an important QT-modifying mechanism. Deciphering the mechanisms that regulate dynamic I_Ks_ subunit stoichiometry in vivo may serve as the basis for the development of novel antiarrhythmic treatment strategies.

## 4. Methods

### 4.1. Molecular Cloning Procedures

The eukaryotic expression vector plasmid carrying the human KCNQ1 cDNA clone (ON287379) was kindly provided by Dirk J. Snyders [39]. The cloning of the wild type and the LQT5 mutant human KCNE1 cDNA constructs encoding WT-KCNE1 (ON237361) and G52R-KCNE1 (ON237362) carrying the p.Gly52Arg mutation was recently described [12]. In this study, the KCNE1 cDNAs were subcloned in the eukaryotic expression vector pcDNA3.1. The D76N-KCNE1 variant (ON237363), carrying the p.Asp76Asn mutation was generated by introducing the corresponding mutation into the WT-KCNE1- or WT-KCNJ2-encoding cDNA sequence by the overlap-extension PCR technique [40]. The non-conductive KCNJ2 variant R218Q-KCNJ2 (ON237366) carrying the p.Arg218Gln mutation was available from a previous study [22]. The human KCNE2 cDNA clone (ON237364) (Accession: BC112087, Clone ID: 8327555) and the KCNE3 cDNA clone (ON237365) (Accession: BC110612, Clone ID: 40030456) were obtained from the Mammalian Gene Collection (GE Healthcare Dharmacon Inc., Lafayette, CO, USA) and were subcloned into pcDNA3.1. Standard laboratory practices were followed during the molecular cloning experiments. All resulting plasmid constructs were sequence-validated.

### 4.2. Heterologous Expression System

Combinations of KCNQ1- and KCNE-encoding cDNA constructs were heterologously over-expressed in an expression system based on Chinese hamster ovary cells (CHO; ATCC, Manassas, VA, USA). CHO cells were cultured in F12 medium (Lonza, Verniers, Belgium) supplemented with 10% fetal bovine serum (PAA, Paschling, Austria) at 37 °C in humidified atmosphere containing 5% CO_2_.

Transient transfections were carried out as follows. CHO cells were plated one day before transfection in 60 mm diameter culture dishes. A total amount of 6.92 μg cDNA plasmid DNA, purified by Nucleobond PC100 anion-exchange columns (Macherey-Nagel GmbH & Co. KG, Düren, Germany) and 20.76 μg polyethylenimine (PEI, 25 kDa, linear, Polysciences Inc., Warrington, PA, USA) were combined in 1.5 mL serum-free F12. The transfection mixture was incubated at room temperature for 30 min, during which time the cells were washed with serum-free F12 twice. After washing, the transfection mixture was added to the cells and the culture dishes were moved back into the CO_2_ incubator for 2 h. At the end of the incubation period, the transfection mixture was replaced by growth medium. Molecular weight of plasmid constructs were calculated based on the known size of each plasmid construct and was taken into account when calculating DNA amounts in transfections (Table S1). Transfection mixtures for all experimental groups contained equivalent amount of the KCNQ1-encoding plasmid. Any additional ion channel subunit-encoding plasmid was included in an amount that contained two times the copy number of the KCNQ1 plasmid. For example, the transfection mixture for group 2 contained KCNQ1-, WT-KCNE1- and KCNE3-encoding plasmids in a 1:2:2 copy number ratio, whereas in group 6, the copy number ratio of the plasmids for KCNQ1, WT-KCNE1, G52R-KCNE1 and KCNE3 was 1:2:2:2. A green fluorescent protein (GFP)-expressing plasmid was also included in each transfection to equalize the DNA load in and also to allow identification of successfully transfected cells during patch clamp experiments. 

### 4.3. Electrophysiology

Whole-cell voltage clamp experiments on transiently transfected CHO cells were carried out 40–48 h post-transfection using the Axopatch 200B patch clamp amplifier and the Digidata 1550 interface, controlled by the pClamp 10 software package (Molecular Devices). Membrane currents were sampled at 1 kHz without filtering. The external solution contained (in mmol/L): NaCl 144, NaH_2_PO_4_ 0.4, KCl 4, MgSO_4_ 0.53, CaCl_2_ 1.8, glucose 5.5, and HEPES 5; pH was adjusted to 7.4 with NaOH. Electrodes were filled with a solution containing (in mmol/L): KOH 110, KCl 40, K2ATP 10, HEPES 5, EGTA 5, and MgCl_2_ 0.1; pH was adjusted to 7.2 with aspartic acid. The measurements were carried out at 37 °C. Cell capacitance was estimated by integrating capacitive transients evoked by hyperpolarizing voltage steps, series resistance was routinely compensated up to 70–80%. Current amplitudes were decreasing substantially after establishment of whole-cell configuration (i.e., rupture of membrane patch beneath the recording electrode) in most experiments. This process was monitored by activating KCNQ1-based currents by applying 5-s-long 50 mV voltage steps from a holding potential of −80 mV at 0.05 Hz. When current amplitudes stabilized (typically 2 to 3 min following brake-in), KCNQ1-based currents were elicited by 5 s long depolarizing potentials between 50 and −20 mV, arising from the holding potential of −80 mV, followed by a repolarizing step to −40 mV to record deactivating tail currents. Current densities were calculated by normalizing the peak of tail currents by the cell capacitance. Deactivation kinetics were assessed by two independent ways, by observing half decay times as defined by the time from tail current peak to 50% decay and by fitting deactivating tail currents by the biexponential function defined by Equation (1):

(1) $$f\left(t\right)=\overset{2}{\underset{i=1}{\sum }}A_{i}e^{-\frac{t}{\tau_{i}}}+C$$

where *t* is time; *C* is constant; *A_i_* are amplitudes and *τ_i_* are time constants of exponential components. The time constant of current activation was obtained from the analysis of recordings made by using the envelope of tail currents protocol, where 50 mV pulses of variable length ranging 100 to 5000 ms were followed by a step back to −40 mV. Peak tail currents were normalized to the maximum tail current amplitude, were plotted as a function of pulse duration and were fitted with an exponential function given in Equation (2):

(2) $$f\left(t\right)=Ae^{-\frac{t}{\tau }}+C$$

where *t* is time; *C* is a constant; *A* is amplitude and *τ* is a time-constant. Steady-state activation curves were established by calculating relative conductance defined as tail current divided by the driving force and normalized to maximal conductance and plotting against the test pulse voltage. Activation curves were fitted by the Boltzmann function, defined by Equation (3):

(3) $$f\left(V\right)=\frac{G_{max}}{1+e^{\frac{V_{1/2}-V}{k}}}$$

where *G* is conductance; *V* is test pulse voltage; *V*_1/2_ is voltage of half-maximal activation and *k* is slope factor. Current recordings were analyzed off-line and non-linear regression was carried out by using Clampfit software (version 10.3.1.5, Molecular Devices).

### 4.4. NanoBiT Protein: Protein Interaction Assay

Co-assembly of KCNQ1 and KCNE1 in the presence and absence of KCNE3 was assessed by using the NanoBiT Protein: Protein Interaction System (Promega, Madison WI, USA), following the manufacturer’s instructions. KCNQ1 and KCNE1 cDNAs were subcloned in the expression vectors supplied with the kit, encoding the large or the small fragment of the Nanoluc luciferase (LgBiT and SmBiT, respectively) by standard laboratory procedures, yielding plasmid constructs encoding the C-terminal fusion of KCNQ1 and LgBiT (KCNQ1-LgBiT) and KCNE1 and SmBiT (KCNE1-SmBiT) and were sequence-verified. Human embryonic kidney cells (HEK293) were cultured in DMEM:F12 medium (Lonza, Verviers, Belgium) supplemented with 10% FBS (EuroClone, Pero (MI), Italy) at 37 °C in humidified atmosphere containing 5% CO_2_. 3.2 × 10^6^ HEK cells were plated in 6 cm culture dishes on the day before the transfection. Transfection mixtures contained 26.7 µg PEI and 8.9 µg DNA in total, containing 2 µg KCNQ1-LgBiT, 2.6 µg KCNE1-SmBiT corresponding to a 1:2 KCNQ1: KCNE1 cDNA copy number ratio. In addition, while the amount of KCNQ1 and KCNE1 cDNA was kept constant, varying the amount of the plasmid encoding the unlabeled KCNE3 in was added resulting in 2:0, 2:1 or 2:2 KCNE1: KCNE3 cDNA copy number ratio. The R218Q-KCNJ2-encoding plasmid was used to keep the total DNA amount constant in all experimental groups. Then, 48 h post-transfection, 1.6 × 10^5^ cells were loaded in 100 µL serum-free DMEM:F12 medium (Lonza, Verviers, Belgium) per well in a 96-well plate. A five-fold working solution of the NanoGlo Live Cell Reagent (Promega, Madison, WI, USA) was prepared in a reduced light environment and 25 µL of it was added to the cells per well. The plate was then moved into a FLUOstar Optima Microplate Reader (BMG Labtech, Ortenberg, Germany, time = 0 min) and was read in luminescence mode. Luminescence values were collected after a 22 min long incubation period and was used for statistical analysis. A total of 6 independent transfections were carried out on separate days including triplicates for each experimental group. Each reported data point represents the average of the three corresponding technical replicates.

### 4.5. Statistics

Data are presented as mean ±95% confidence interval of the mean (95% CI). Group means from the patch clamp analysis were compared by one-way ANOVA and by ANOVA for correlated samples in the protein: protein interaction assay. Post-hoc tests with correction for multiple comparisons were carried out according to the Holm–Sidak method. Statistical tests were computed by using the GraphPad Prism software (version 8, GraphPad Software Inc., San Diego, CA, USA).

## Data Availability

Sequence data of plasmids used in the current study are available under the following GenBank accession numbers: KCNQ1_pIRES2-EGFP ON287379, S38G-KCNE1 ON237361, G52R-KCNE1 ON237362, D76N-KCNE1 ON237363, KCNE2 ON237364, KCNE3 ON237365 and R218Q-KCNJ2 ON237366. Data is contained within the article and Supplementary Material.

## Supplementary Materials

The following supporting information can be downloaded at: https://www.mdpi.com/article/10.3390/ph15121550/s1, Figure S1: Time constants of the fast and slow exponential components (activation kinetics).; Figure S2: Time constant of the slow exponential component (deactivation kinetic).; Table S1: Plasmid DNA amount used in co-transfection experiments for patch clamping.
