# Supplementary material for: A Possible Explanation for the Low Penetrance of Pathogenic KCNE1 Variants in Long QT Syndrome Type 5
Source: Pharmaceuticals (Basel). 2022 Dec 13;15(12):1550. doi: 10.3390/ph15121550 (PMC9782992; doi:10.3390/ph15121550)
Supplement: Supplementary file 1 [file pharmaceuticals-15-01550-s001.zip › pharmaceuticals-2050539-supplementary.pdf]

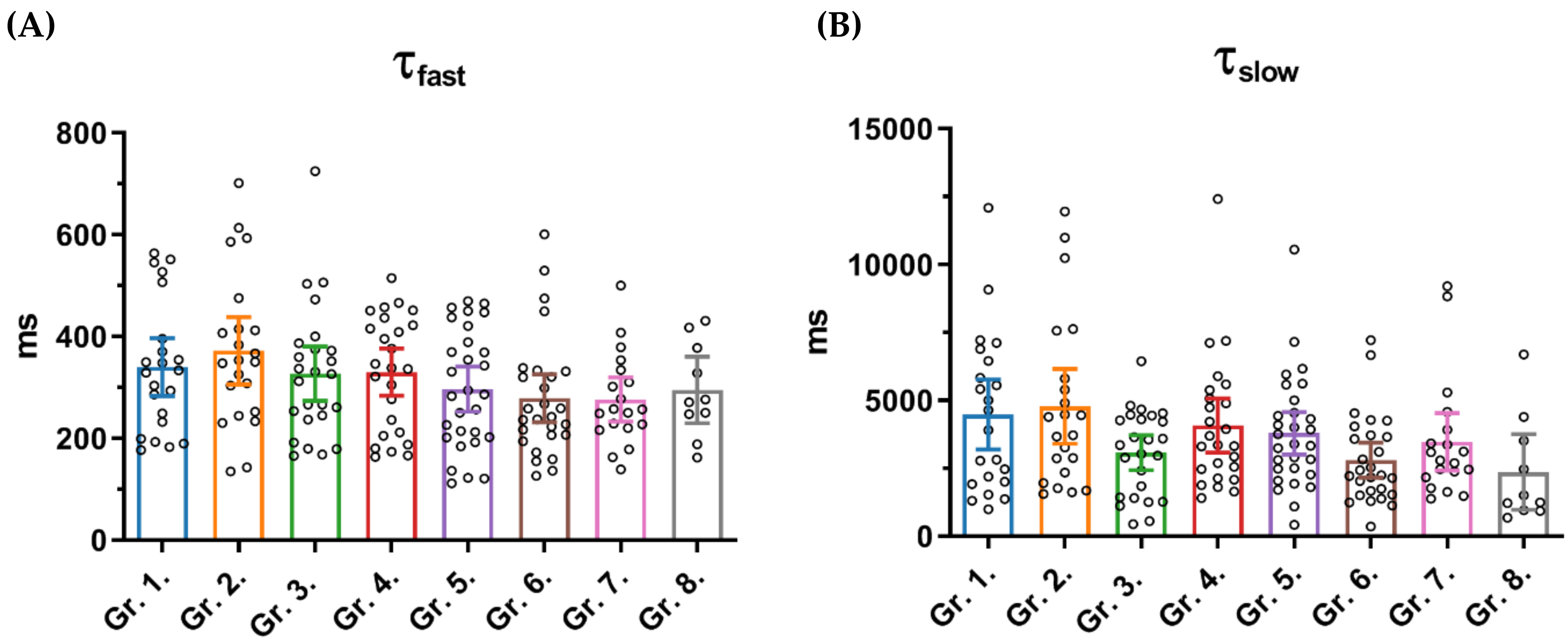

Figure S1. Time constants of the fast (A) and slow (B) exponential components extracted from non-linear curve fits of activating current traces. Symbols represent individual data points, bar graph represent mean ± 95% CI.

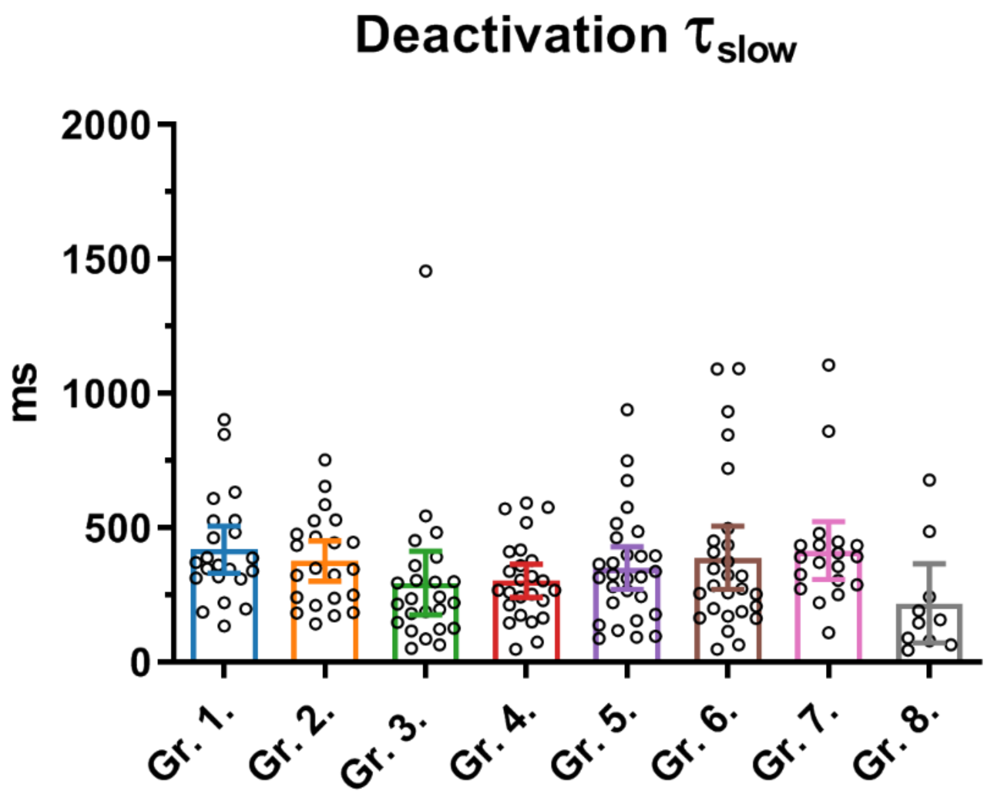

Figure S2. Time constant of the slow exponential component extracted from non-linear curve fits of tail current traces. Symbols represent individual data points, bar graph represent mean ± 95% CI. Group means were statistically compared by one-way ANOVA followed by Holm-Sidak`s post-hoc tests.

Table S1. Plasmid DNA amount used in co-transfection experiments for patch clamping.

Group		Plasmid construct (μg)							
		GFP	KCNQ1	WT-KCNE1	G52R-KCNE1	D76N-KCNE1	KCNE2	KCNE3	R218Q-KCNJ2
Gr. 1.:	KCNQ1 + WT-KCNE1	4.32	1	1.6	-	-	-	-	-
Gr. 2.:	KCNQ1 + WT-KCNE1 + KCNE3	2.6	1	1.6	-	-	-	1.72	-
Gr. 3.:	KCNQ1 + WT-KCNE1 + D76N-KCNE1	2.72	1	1.6	-	1.6	-	-	-
Gr. 4.:	KCNQ1 + WT-KCNE1 + D76N-KCNE1 + KCNE3	1	1	1.6	-	1.6	-	1.72	-
Gr. 5.:	KCNQ1 + WT-KCNE1 + G52R-KCNE1	2.72	1	1.6	1.6	-	-	-	-

Gr. 6.:	KCNQ1 + WT-KCNE1 + G52R-KCNE1 + KCNE3	1	1	1.6	1.6	-	-	1.72	-
Gr. 7.:	KCNQ1 + WT-KCNE1 + G52R-KCNE1 + KCNE2	1.09	1	1.6	1.6	-	1.63	-	-
Gr. 8.:	KCNQ1 + WT-KCNE1 + D76N-KCNE1 + R218Q-kCNJ2	0.89	1	1.6	-	1.6	-	-	1.83
